# Research protocol of two concurrent cluster-randomized trials: Real-life Effect of a CAMPaign with Measles Vaccination (RECAMP-MV) and Real-life Effect of a CAMPaign with Oral Polio Vaccination (RECAMP-OPV) on mortality and morbidity among children in rural Guinea-Bissau

**DOI:** 10.1186/s12889-019-7813-y

**Published:** 2019-11-11

**Authors:** A. Varma, A. K. G. Jensen, S. M. Thysen, L. M. Pedersen, P. Aaby, A. B. Fisker

**Affiliations:** 1 Department of Clinical Research, OPEN, University of Southern Denmark, Winsløwparken 19, 5000 Odense C, Denmark; 2 Bandim Health Project, Indepth Network, Apartado 861, 1004 Bissau Codex, Guinea-Bissau; 3 Research Center of Vitamins and Vaccines, Statens Serum Institut, Bandim Health Project, Artillerivej 5, 2300 Copenhagen, Denmark; 4 Section of Biostatistics, University of Copenhagen, Øster Farimagsgade 5, 1014 Copenhagen K, Denmark; 5 Department of Public Health, University of Aarhus, Bartholins Alle 2, 8000 Aarhus C, Denmark

## Abstract

**Background:**

Measles and oral polio vaccinations may reduce child mortality to an extent that cannot be explained by prevention of measles and polio infections; these vaccines seem to have beneficial non-specific effects. In the last decades, billions of children worldwide have received measles vaccine (MV) and oral polio vaccine (OPV) through campaigns. Meanwhile the under-five child mortality has declined. Past MV and OPV campaigns may have contributed to this decline, even in the absence of measles and polio infections. However, cessation of these campaigns, once their targeted infections are eradicated, may reverse the decline in the under-five child mortality. No randomized trial has assessed the real-life effect of either campaign on child mortality and morbidity. We present the research protocol of two concurrent trials: RECAMP-MV and RECAMP-OPV.

**Methods:**

Both trials are cluster-randomized trials among children registered in Bandim Health Project’s rural health and demographic surveillance system throughout Guinea-Bissau. RECAMP-MV is conducted among children aged 9–59 months and RECAMP-OPV is conducted among children aged 0–8 months. We randomized 222 geographical clusters to intervention or control clusters. In intervention clusters, children are offered MV or OPV (according to age at enrolment) and a health check-up. In control clusters, children are offered only a health check-up. Enrolments began in November 2016 (RECAMP-MV) and March 2017 (RECAMP-OPV). We plan 18,000 enrolments for RECAMP-MV with an average follow-up period of 18 months and 10,000 enrolments for RECAMP-OPV with an average follow-up period of 10 months. Data collection is ongoing. The primary outcome in both trials is non-accidental death or non-accidental first non-fatal hospitalization with overnight stay (composite outcome). Secondary outcomes are: non-accidental death, repeated non-fatal hospitalizations with overnight stay, cause-specific primary outcome, outpatient visit, and illness. We obtained ethical approval from Guinea-Bissau and consultative approval from Denmark.

**Discussion:**

Cluster randomization and minimum risk of loss to follow-up are strengths, and no placebo a limitation. Our trials challenge the understanding that MV and OPV only prevent measles and polio, and that once both infections are eradicated, campaigns with MV and OPV can be phased out without negative implications on child health and survival.

**Trial registration:**

NCT03460002.

## Background

The common public health understanding is that vaccines protect against their target infections and do little else. However, an increasing body of evidence challenges this understanding. Studies from low-income countries suggest that the live measles vaccine (MV) and the live oral polio vaccine (OPV) reduce the under-five child mortality to an extent that cannot be explained by prevention of measles or polio infections; both vaccines seem to have what is termed beneficial non-specific effects (NSEs) [1]. In a systematic review commissioned by the World Health Organization’s (WHO) Strategic Advisory Group of Experts on Immunizations (SAGE) it was concluded in 2014 that, “There was consistent evidence of a beneficial effect of measles vaccine (…)” on child mortality. This conclusion was based on four randomized trials and 18 observational studies [2] but SAGE called for more research [3]. Though the effect of OPV was not included in the review, OPV also seems to have beneficial NSEs: In two trials, where infants were randomised to OPV or no OPV at birth, OPV was associated with a 32% lower infant mortality in both trials [4, 5]. Observational studies also indicate that children receiving OPV with a non-live vaccine have better survival than children receiving the non-live vaccine only [6, 7]. We respond to SAGE’s call for more research by assessing the NSEs of an MV campaign and an OPV campaign.

In the last decades, billions of children have received MV and OPV through campaigns implemented worldwide with the goal to ultimately eradicate measles and polio infections [8, 9]. The campaigns aim at reaching all children in a broad age-group regardless of pre-campaign vaccination status, also children who are not reached through routine vaccination program services. The campaigns can increase population immunity against the viruses rapidly, thereby interrupting virus transmission, which leads to herd protection [10, 11]. The under-five child mortality has declined on a global scale [12] in the same period as campaigns with MV and OPV have been conducted. Thus, we suggest that past MV and OPV campaigns may have efficiently contributed to reduce the under-five child mortality given their presumed beneficial NSEs, even in the absence of measles and polio infections. Cessation of MV and OPV campaigns after eradication of their targeted infections may therefore reverse the declining trend in the under-five child mortality.

No randomized trial has assessed the real-life effect of an MV campaign or an OPV campaign on child mortality. Two observational studies were published after WHO’s SAGE review, and both support that MV campaigns among children reduced the under-five mortality. In a before/after study among 8000 children, mortality in the year following an MV campaign was 20% (4–34%) lower compared with the year prior to the campaign, even after censoring measles deaths (17% (0–31%)) [13]. Another study compared mortality in MV campaign participants (5633) with non-participants (1006) in the year following the campaign, and mortality was found to be 72% (23–90%) lower among participants; no deaths were measles related [14]. In both studies, children who were also measles vaccinated through routine vaccination program services seemed to additionally benefit from the MV campaign [13, 14]. Similarly, OPV campaigns may reduce child mortality considerably. Among children followed in seven trials of vaccines or vitamin A supplements, an OPV campaign was associated with a 19% (5–32%) lower mortality rate [15]. Comparisons of mortality after OPV campaigns with mortality before the campaigns in other cohorts also indicated lower mortality for children exposed to OPV campaigns [16, 17], and adjusted for age, receiving OPV in a campaign was associated with a 91% (20–99%) lower mortality than not receiving OPV in a campaign [18].

In two concurrent cluster-randomized controlled trials, we want to assess the separate effect of an MV campaign and an OPV campaign on child health and survival in the absence of measles and polio infections. We present the research protocol of each trial: Real-life Effect of a CAMPaign with a Measles Vaccination (RECAMP-MV) and Real-life Effect of a CAMPaign with an Oral Polio Vaccination (RECAMP-OPV). We initiated enrolments into RECAMP-MV in November 2016 and will conduct follow-up until eligibility for a national measles vaccination campaign (trial completion is expected by late 2019). We initiated enrolments into RECAMP-OPV in March 2017 and will conduct follow-up until eligibility for any national vaccination campaign or a maximum of 12 months (trial completion is expected by late 2020). A data safety and monitoring board consisting of a statistician, a pediatrician, and an epidemiologist is providing input to ensure an optimal data collection process.

### Objectives

#### Primary objectives

RECAMP-MV: To assess the real-life effect of an MV campaign among children aged 9–59 months on non-accidental mortality or non-accidental morbidity (composite outcome) in rural Guinea-Bissau, where measles infection is limited. We will test whether an MV campaign can reduce the composite outcome by 30% during an average follow-up period of 18 months.

RECAMP-OPV: To assess the real-life effect of an OPV campaign among children aged 0–8 months on non-accidental mortality or non-accidental morbidity (composite outcome) in rural Guinea-Bissau, where no polio circulates. We will test whether an OPV campaign can reduce the composite outcome by 25% during an average follow-up period of 10 months.

#### Secondary objectives

To better understand the real-life effect of either campaign on child health and survival we will also assess the effect of each campaign on other health measures and under different scenarios as specified in the statistical analysis section.

## Methods/design

### Setting

Guinea-Bissau’s Ministry of Health has implemented national MV campaigns among children aged 9–59 months every third year since 2006 [19], and OPV campaigns more frequently [15]. Despite some fluctuations in reported measles infection cases [20] and MV coverage [21], Guinea-Bissau has a low risk profile of measles (Table 1) and the last recorded case of indigenous wild poliovirus in Guinea-Bissau was in 1999 [22].

**Table 1:** Measles infection cases, vaccination coverage and vaccination campaigns in Guinea-Bissau according to WHO’s country data [20, 21]

*Year*	2008	2009*	2010	2011	2012*	2013	2014	2015*	2016	2017
Measles infection cases (number)	12	0	26	0	0	0	1	153	0	11
1st routine measles dose (proportion)	64%	79%	78%	78%	90%	89%	81%	90%	71%	66%

*Guinea-Bissau’s Ministry of Health implemented a national measles vaccination campaign

*Abbreviation*: WHO=World Health Organization

Bandim Health Project (BHP) follows women of fertile age and children under-five in Guinea-Bissau’s rural population through a health and demographic surveillance system (HDSS). This enables assessment of child mortality and morbidity. In Guinea-Bissau’s nine rural health regions (Oio, Biombo, Gabu, Cacheu, Bafata, Quinara, Tombali, Bubaque, and Bolama), 222 randomly selected geographical clusters with more than 22,000 children under-five are being monitored. The selection process of the geographical clusters has been described elsewhere [23]. Field teams conduct regular visits to all villages in all clusters. At household visits field assistants register pregnancies, and children’s vaccination status, mortality, morbidity, nutritional status, campaigns with other health interventions, migration, and whereabouts if absent [23]. This is the implementation platform of RECAMP-MV and RECAMP-OPV.

### Design and randomization

The 222 village clusters were randomized to intervention or control clusters stratified by region and access to health services. We defined health service access as vaccination coverage by 12 months of age assessed among children aged 12–23 months [24], using BHP HDSS data from 2015 to 2016. This pre-trial vaccination coverage was based on Bacillus Calmette Guerin (BCG) vaccine, three doses of OPV, three doses of Pentavalent (diphtheria, tetanus, pertussis, hepatitis B, haemophilus influenza type B), and MV. We defined low and high pre-trial vaccination coverage using the median as a cut-off point. Within each of the two coverage strata per region, we assigned one half of the clusters to receive intervention and health check-up, and the other half to receive only health check-up, based on an externally generated random number.

### Study population

Children living in BHP’s rural HDSS are eligible to enter RECAMP-MV if 9–59 months of age and RECAMP-OPV if 0–8 months of age. A child is excluded if it: 1) is considered overtly ill by the enrolling nurse or 2) has an axillary temperature > 39 °C or 3) is aged > 6 months and has a mid-upper-arm-circumference < 110 mm or 4) has experienced an allergic reaction after a prior vaccination or 5) is followed in another ongoing BHP trial in rural Guinea-Bissau (i.e. children from RECAMP-OPV will not be enrolled into RECAMP-MV once they turn 9 months old, and children who are < 2 months old and enrolled in a randomized trial giving BCG and OPV at a home visit shortly after birth [25] will not be enrolled into RECAMP-OPV). Criteria 1–4 ensure that we avoid enrolment of severely acutely ill or immunocompromised children, and these criteria are based on WHO recommendations [10, 11], which we have translated into local practice. Criteria 5 is set to avoid data interpretative issues.

### Intervention

RECAMP-MV: We offer one dose of a WHO pre-qualified monovalent live attenuated measles vaccine (Edmonston-Zagreb strain from Serum Institute of India) to children aged 9–59 months in intervention clusters.

RECAMP-OPV: We offer one dose of a WHO prequalified standard bivalent OPV to children aged 0–8 months in intervention clusters. To mimic the way most OPV campaigns are implemented, we initiated the RECAMP-OPV trial with two visits one month apart where logistically feasible. At the second visit, a second dose of OPV is offered to children in intervention clusters, while children in both control and intervention clusters are health examined and weighed.

**Fig. 1:**
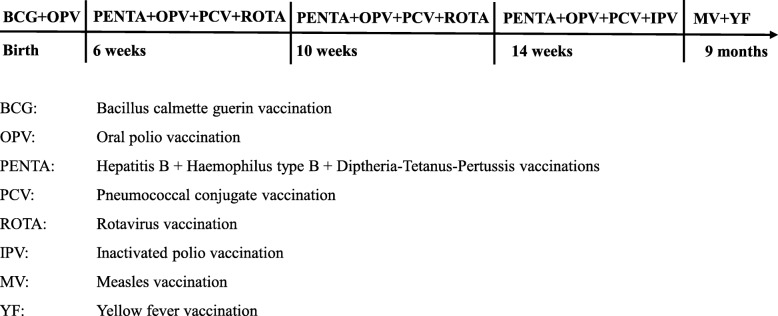
Guinea-Bissau’s routine child vaccination program

The cold chain for both vaccines is documented. We provide the MV and OPV campaign vaccinations independently from Guinea-Bissau’s routine child vaccination program [26] (Fig. 1).

### Blinding

It is common practice in Guinea-Bissau that when a mother/guardian visits the health system with a sick child, the mother/guardian brings the child’s vaccination card; it could be speculated that health system personnel give low treatment priority to children with high vaccination coverage and vice versa. Thus, to avoid the risk of differential treatment decision by health system personnel, we do not register cluster assignment on the children’s vaccination cards.

### Sample size considerations

RECAMP-MV: Children are followed from enrolment and until eligibility for a national MV campaign. We aim to have minimum 80% power to detect at least a 30% reduction in non-accidental mortality or non-accidental morbidity (composite outcome) given that this is the true reduction during an average follow-up period of 18 months. The planned sample size was originally based on 182 clusters and a composite outcome rate of 20/1000 person-years among children aged 9–59 months in BHP’s rural HDSS. Based on Hayes and Moulton’s power formula for a cluster-randomized trial [27], our initial power calculations indicated 86% power to detect at least a 30% reduction if we enrolled 14,500 children (with a between cluster variation coefficient of 0.25 and a harmonic mean (reciprocal of the arithmetic means of the reciprocals) of total projected accumulated observation time per cluster of 107 person-years at risk). After discussions with our data safety and monitoring board we decided to re-evaluate the power calculations for both trials when more information on outcome rates and distribution of enrolments between the clusters was gained, to verify total enrolments needed. This re-evaluation was based on data from the first complete round of enrolment and follow-up visit. As Table 2 shows for RECAMP-MV, we observed a lower composite outcome rate and harmonic mean of total projected accumulated observation time per cluster, than expected. Our data safety and monitoring board supported our decision to enlarge the number of clusters from 182 to 222. This increased the planned enrolments from 14,500 to 18,000 children which gives us 80% power to detect a 30% reduction in the composite outcome (with an assumed control cluster outcome rate of 17/1000 person-years at risk and a harmonic mean of total projected accumulated observation time per cluster of 84 person-years at risk).

**Table 2:** Sample size estimates derived from the first complete round of enrolment and follow-up visit of RECAMP-MV and RECAMP-OPV

RECAMP	MV	OPV
Number of clusters	222	
Alpha	0.05	
Between cluster variation coefficient	0.25	
Number of eligible children to be enrolled	18,000	10,000
Observed non-accidental deaths/non-accidental hospitalizations rates	15/1000 pyrs*	48/1000 pyrs**
Harmonic mean of total projected accumulated observation time per cluster	84 pyrs	40 pyrs
Expected reduction	30%	25%
*Power*	*80%*	*80%*

*Assuming that our observed composite outcome rate (15/1000 pyrs) is an average of the rates in our control and intervention clusters, and that the real difference between the clusters is 30%, we assumed the rates to be 17/1000 pyrs in control clusters and 12/1000 pyrs in intervention clusters when re-evaluating our power calculations

**Assuming that our observed composite outcome rate (48/1000 pyrs) is an average of the rates in our control and intervention clusters, and that the real difference between the clusters is 25%, we assumed the rates to be 55/1000 pyrs in control clusters and 41/1000 pyrs in intervention clusters when re-evaluating our power calculations

*Abbreviation*: Pyrs = person-years at risk

RECAMP-OPV: Children are followed from enrolment and until eligibility for any national vaccination campaign or for a maximum of 12 months. We aim to have minimum 80% power to detect at least a 25% reduction in non-accidental mortality or non-accidental morbidity (composite outcome) given that this is the true reduction during an average follow-up period of 10 months. The planned sample size was originally based on 182 clusters and a composite outcome rate of 70/1000 person-years among children aged 0–8 months in BHP’s rural HDSS. Based on Hayes and Moulton’s power formula for a cluster-randomized trial [27], our initial power calculations indicated 80% power to detect at least a 25% reduction if we enrolled 6500 children (with a between cluster variation coefficient of 0.25 and a harmonic mean (reciprocal of the arithmetic means of the reciprocals) of total projected accumulated observation time per cluster of 40 person-years at risk). After the re-evaluation we observed a lower composite outcome rate than expected. We enlarged the number of clusters from 182 to 222 and increased the planned number of enrolments from 6500 to 10,000 children which gives us 80% power to detect a 25% reduction in the composite outcome (with an assumed control cluster outcome rate of 55/1000 person-years at risk and a harmonic mean of total projected accumulated observation time per cluster of 40 person-years at risk).

Table 2 summarizes the final sample size calculation estimates for both trials.

### Enrolment and follow-up procedures

A pilot phase was initiated in Biombo from November 2016 to March 2017. We trained three field teams, each consisting of at least four field assistants and one enrolling nurse. The consent process, structured interviews during enrolment, and structured interviews during follow-up take place in Portuguese Creole managed by the field teams (interview questions are written in Portuguese and based on BHP’s rural HDSS questionnaires used in previous studies). If necessary, a villager is called to act as a translator.

For RECAMP-MV, we plan 2–3 enrolment rounds to visit children who were not home at a previous enrolment visit, or who later move into BHP’s rural HDSS area. For RECAMP-OPV more enrolment rounds are needed. The written consent process is two phased: 1) a field assistant conducts a household visit where he explains to the mother/guardian of an eligible and present child that, “In the past the Ministry of Health has provided many MV and OPV campaigns as the mother/guardian probably remembers. Now there is rarely any measles infection and no polio infection in Guinea-Bissau. Therefore, in the future, the campaigns may stop. BHP wants to know if it is good for children’s health to stop or continue MV and OPV campaigns. To know this, we will vaccinate in some but not other villages. When the work is done all children aged >9 months in the villages that do not receive vaccines today will be offered MV. If you are interested your child should be brought to our health post today”, the field assistant does not inform the mother/guardian about cluster assignment when referring to the health post. 2) at the health post an enrolling nurse/field assistant carefully explains both trials, usually to several mothers/guardians at a time. If a mother/guardian is illiterate a witness independent from the field team is called. After the explanation, any questions from the mothers/guardians are welcomed. If mothers/guardians want their children to participate they are requested to give their signatures/fingerprints on a consent form and then they receive an information letter written in Portuguese.

After consent, the enrolling nurse performs a health check-up of one child at a time (assessing illness, issues with prior vaccination, and measuring axillary temperature, mid-upper-arm-circumference, and weight). If the enrolling nurse experiences that a child is overtly ill, the child’s mother/guardian is given health advice, and if necessary offered health facility transport, irrespective of cluster assignment. Overtly ill children are offered enrolment at a subsequent visit, if recovered.

If a child is assessed healthy in intervention clusters, the child aged 9–59 months is administered a 0.5 ml reconstituted MV from a 10-dose vial by deep subcutaneous injection into the left subscapular region (leftover doses are discarded six hours after reconstitution), and the child aged 0–8 months is administered two oral drops of OPV from a multi-dose vial (leftover doses are discarded after 28 days). If a child is assessed healthy in control clusters, the child is not administered MV or OPV.

**Fig. 2:**
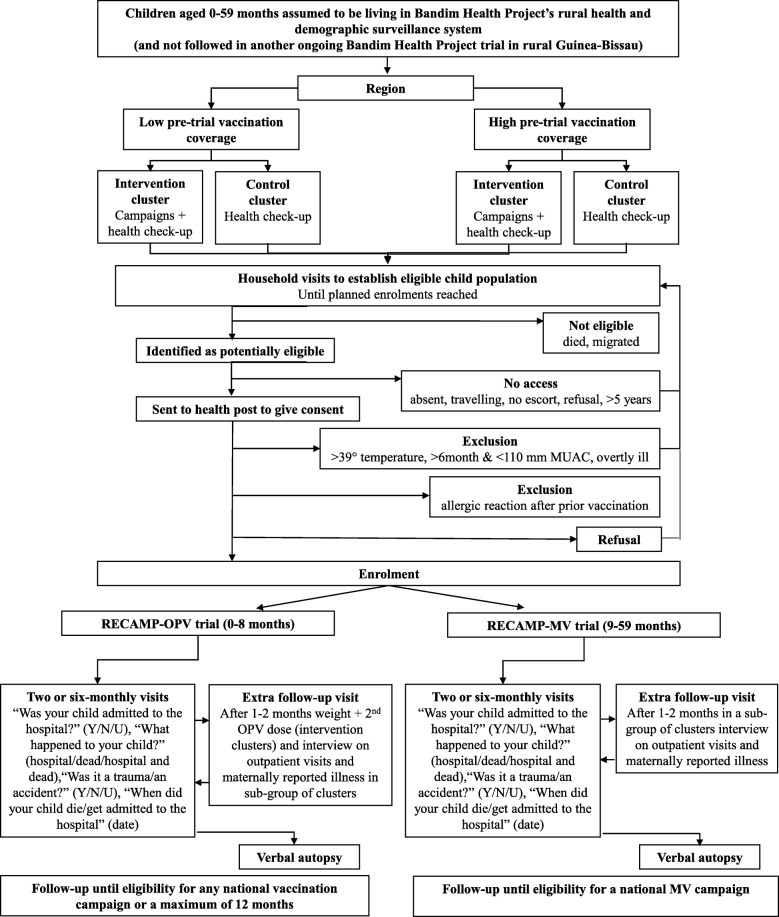
Flowchart from eligibility to follow-up in RECAMP-MV and RECAMP-OPV. Abbreviations: MV = measles vaccination; OPV = oral polio vaccination; MUAC = mid-upper-arm circumference; Y/N/U = yes/no/unknown

All enrolled children are followed through regular household visits by field assistants who collect information on death and hospitalization, among other. The visits are conducted every two months to children < 12 months of age in Oio, Biombo and Cacheu for logistical reasons, and every six months to older children, and in the remaining regions. If a field assistant registers the death of a child, a specially trained field assistant conducts a verbal autopsy at a subsequent visit [28]. An extra follow-up visit is conducted among a subgroup of enrolled children one-two months after enrolment; the mothers/guardians of these children are visited by field assistants to collect information on outpatient visit and maternally reported illness in the elapsed time span. This visit is also utilized to provide a second OPV dose to children aged 0–8 months in intervention clusters. Figure 2 shows the flow from eligibility to follow-up in RECAMP-MV and RECAMP-OPV.

### Outcomes

The primary outcome for each trial is defined as a composite outcome to ensure sufficient power. It consists of:

- non-accidental mortality or
- non-accidental morbidity (first non-fatal hospitalization with overnight stay)

The secondary outcomes assess other health measures. They consist of:

- non-accidental mortality
- non-accidental repeated morbidity (at least one non-fatal hospitalization with overnight stay)
- cause-specific primary outcome (malaria, diarrhea, and respiratory infections [29])
- proportion of non-accidental outpatient visits and non-accidental maternally reported illnesses in a sub-group 1–2 months after enrolment in the elapsed time span
- Furthermore in RECAMP-OPV: weight at the extra follow-up visit 1–2 months after enrolment

### Adverse reactions

RECAMP-MV: Common mild adverse reactions to MV include injection site reactions (within 24 h), fever (within 7–12 days), or rash (within 7–10 days); all resolve within 1–3 days. Except from febrile seizures, severe adverse reactions are extremely rare (anaphylaxis, thrombocytopenia, and encephalomyelitis) [30]. MV campaigns’ safety profile has been evaluated, and severe adverse reactions seem rare [31–35].

RECAMP-OPV: Common mild adverse reactions to OPV include self-limiting diarrhoea [36]. The only severe adverse reaction is vaccine derived polio, which occurs in 2–4 infants in a birth cohort of one million children receiving 4 doses of OPV during the first months of life [11]. The risk of circulating vaccine derived polio has been markedly lowered with the shift from trivalent (type 1–2-3) to bivalent OPV (type 1 and 3) [37], as 94% of the circulating vaccine derived polio was caused by type 2 [38].

To the extent possible, we are in contact with the adverse events following immunization (AEFI) responsible from each region to register any potential adverse reaction caused by our campaigns.

### Data management

Data collected by the field teams is transported back to Bissau regularly. Data entry assistants enter the data in DBASE, and they also clean the data based on pre-specified cleaning programs. Data collected during the pilot phase will be included in the analyses to obtain sufficient power. Through crosslinks with other data sources, key variables are verified and data entry errors are captured.

### Statistical analyses

Statistical analyses will be performed in STATA by the research group based on data analysis plans which have been reviewed by the data safety and monitoring board (Additional file 1 (RECAMP-MV) and Additional file 2 (RECAMP-OPV)). If further analyses are planned due to new knowledge arising during the trials, potential amendments to the respective data analysis plan will be discussed with the data safety and monitoring board. We will analyse all primary and secondary outcomes based on individual level data as the cluster size varies. We will present confidence intervals of 95%. Absolute numbers of missing values will be presented, when relevant. No missing values will be imputed. No corrections will be made for multiple testing.

#### Primary analysis of the primary outcome

The main conclusion in RECAMP-MV and RECAMP-OPV will be based on a per-protocol analysis. The primary outcome will be analyzed in a Cox proportional hazards model, adjusted by region, pre-trial vaccination coverage, and sex, with age as the underlying timescale. We will use cluster-robust standard errors to account for intra-cluster correlation. Children will enter the analysis on the day of enrolment, and their follow-up will be censored at:

- RECAMP-MV: death due to accident, migration or eligibility for a national MV campaign, whichever comes first.
- RECAMP-OPV: death due to accident, migration, eligibility for any national vaccination campaign, or a maximum of 12 months of follow-up, whichever comes first.

In both trials, hospital admissions due to accidents are ignored but the follow-up time is censored while the child is admitted.

#### Secondary outcomes

In per-protocol analyses, we will assess the secondary outcomes: non-accidental mortality, non-accidental repeated morbidity, and cause-specific primary outcome. These secondary outcomes will be analyzed in Cox proportional hazards models as described for the primary outcome. Furthermore, we will analyse outpatient visits and maternally reported illnesses occurring 1–2 months after enrolment in per-protocol analyses using log-binomial regression models adjusted for region, pre-trial vaccination coverage, and sex. In RECAMP-OPV, we will furthermore analyze weight and MUAC 1–2 months after enrolment using multiple regression models adjusted for region, pre-trial vaccination coverage, and sex.

#### Effect modifier analyses of primary outcome

In per-protocol analyses we will assess potential effect modifiers of the primary outcome (Tables 3 and 4).

**Table 3:** RECAMP-MV - potential effect modifiers

*Effect modifiers*	*Rationale*
Prior MV status	Prior MV administration may lead to a larger reduction in non-accidental mortality/non-accidental morbidity than no prior MV administration [13, 14, 39, 40]
Sex	Girls may experience a larger reduction in non-accidental mortality/non-accidental morbidity than boys [40–43]
Season	Enrolment in the dry season may lead to a larger reduction in non-accidental mortality/non-accidental morbidity than enrolment in the rainy season [29, 44]
Campaigns with other health interventions	Vitamin A may amplify a beneficial non-specific effect [45]. Inactivated meningitis A and inactivated polio vaccination [42], and oral polio vaccination [17, 46], may neutralize/invert a between cluster difference. Participation status will be assigned on an ecological level, assuming that children eligible for campaigns with other health interventions receive these interventions on the campaign dates.

**Table 4:** RECAMP-OPV - potential effect modifiers

*Effect modifiers*	*Rationale*
Sex	Previous studies have demonstrated that the effect of OPV is stronger in boys than girls [4, 15, 47].
One vs two doses (2nd dose 1 month after enrolment)	Observational studies indicate that additional doses of OPV offer additional benefits [15].
Age at first dose of OPV	A prior study has indicated that the effect of subsequent vaccines may vary with the age at which the gut was primed [16].
Season of enrolment	Some interventions (eg MV and Vitamin A) have stronger effects when given in the dry season [29, 48]. We will investigate whether the effect of OPV varies for children enrolled in the dry season (December–May) versus children enrolled in the rainy season (June–November)
Vitamin A supplements	Vitamin A supplementation may amplify the NSEs of vaccines [49, 50]. We will examine whether the effect of OPV vary before and after being eligible for vitamin A supplementation after enrolment
Prior OPV campaign	Repeated doses of OPV offer additional benefits [15]. If OPV campaigns take place during the study, we will assess whether the effect is similar in children having been exposed/not exposed to prior OPV campaigns.

#### Sensitivity analyses of primary outcome

We will assess the robustness of the primary analysis of the primary outcome for each campaign under different scenarios:

- two intention-to-treat analyses: 1) in a classic intention-to-treat analysis children will be included if present in the cluster from the day they were first potentially eligible to enter but did not because they e.g. did not receive the assigned treatment, were excluded due to illness, did not have a mother/guardian to escort them, or had a mother/guardian who refused participation, 2) in an extended intention-to-treat analysis children will be included if living in the cluster from the day they were first potentially eligible to enter (including children from the classic intention-to-treat analysis and children who were absent/travelling) as either campaign may affect other children’s health in the community by reducing the general infectious pressure.
- Furthermore, we will assess if different censoring criteria affect the robustness of the results

## Ethics and dissemination

Prior to initiating the trials, we obtained ethical approval from Guinea-Bissau’s national ethics committee (Comité Nacional de Ética na Saúde: CNES/2016/020) and consultative approval from Denmark’s national ethics committee (Den Nationale Videnskabsetiske Komité: 1606756). Then we met with all regional health directorates to inform them about the trials’ aim, routines, initiation date, and to request their collaboration. This paper includes amendments resulting from our discussions with the data safety and monitoring board and the sample size enlargements. For these amendments, we obtained ethical approval from Guinea-Bissau’s national ethics committee (Comité Nacional de Ética na Saúde: CNES/2018/028) and consultative approval from Denmark’s national ethics committee (Den Nationale Videnskabsetiske Komité: 1606756). The trials are registered at www.clinicaltrialsgov.com (identifier NCT03460002). Data is being stored according to a general agreement between the BHP, and the Ministry of Health in Guinea-Bissau, and Statens Serum Institut in Denmark. At the BHP’s main office in Guinea-Bissau all questionnaires are physically stored, and databases with enrolment and follow-up information are separately stored at an encrypted server.

We will disseminate the results regardless of positive or negative findings. We intend to publish the results in internationally peer-reviewed journals. We will provide the results to WHO, and Guinea-Bissau’s Primary Health Program, Institute of Public Health and regional health directorates; the Institute of Public Health will receive a copy of the results. Any significant deviations from this paper will be documented in the reported results.

## Discussion

This paper presents the methodology of two concurrent cluster randomized controlled trials named RECAMP-MV and RECAMP-OPV. We will assess the real-life effect of an MV campaign among children aged 9–59 months (RECAMP-MV) and the real-life effect of an OPV campaign among children aged 0–8 months (RECAMP-OPV) on non-accidental mortality or non-accidental morbidity (composite outcome) in rural Guinea-Bissau**,** where measles infection is limited and no polio circulates. Major strengths lie within the cluster randomisation design which allows us to assess each campaign’s effect on the general infectious pressure, which would not be possible with individual randomisation. Furthermore, the BHP’s rural HDSS ensures a reliable and thoroughly tested data collection and data management infrastructure minimizing the risk of loss to follow-up. In the following, we consider a major limitation, circumstantial challenges, and future perspectives.

The major limitation is insufficient blinding. Only blinding health system personnel can make the campaigns appear to have an effect that does not solely depend on the campaigns. However, blinding the research group, field teams and mothers/guardians would require placebo use. Administering another vaccine may trigger NSEs, which could obscure the assessment of the campaigns’ NSEs. Administering saline would be causing many children pain without benefit. However, as death and hospitalization are not subjective, and as their assessment is based on standardized interviews, we expect the risk of differential outcome reporting to be minimized. Though, we do have some speculations about the potential impact of not blinding the mothers/guardians: 1) mothers/guardians in intervention clusters may consider their children as being extra healthy because they have seen their children receive the campaign vaccines. This could make the mothers/guardians less prone to seek help from the health system if their children get ill. Thus, among children whose mothers/guardians state that their children have been ill after enrolment we will assess the proportion of children whose mothers/guardians also state an outpatient visit by cluster assignment. 2) mothers/guardians in intervention clusters could come to know that their children belong to an intervention cluster prior to enrolment because there is no allocation concealment. This could make the mothers/guardians in intervention clusters more motivated to let their children enrol than mothers/guardians in control clusters. Thus, we will assess if there is a difference in the proportion of children whose mothers/guardians choose not to participate for different reasons, by cluster assignment.

We could face some circumstantial challenges. For RECAMP-MV: 1) Guinea-Bissau’s low risk profile makes a measles epidemic seem unlikely. It is nevertheless possible. Our regular contact to the AEFI and disease surveillance responsible in the health regions also ensures registration of suspected measles infection cases. However, declared measles infection cases are likely to be misclassifications of other childhood infections as BHP has experienced in previous studies from Guinea-Bissau. Therefore, only reported measles infection cases when regional health directorates confirm circulating measles will be classified as measles. In such instances, field assistants are instructed to pose questions about symptoms, timing, and source of infection based on BHP’s rural HDSS questionnaires used in previous studies. 2) if Guinea-Bissau’s Ministry of Health does not announce a national MV campaign during the trial, we will conduct a visit to all children enrolled in control clusters to offer them MV after both trials have ended. For RECAMP-OPV: national OPV campaigns implemented during the trial will shorten the follow-up period, which could reduce the power.

In both trials, it may influence enrolment efficiency that we exclude children from other ongoing BHP trials in rural Guinea-Bissau. However, we expect to have included a sufficient number of clusters to avoid under-powering due to this potential challenge.

If RECAMP-MV and/or RECAMP-OPV demonstrate beneficial NSEs of the expected respective magnitudes in the absence of measles and/or polio infections, it will clearly challenge two understandings. Firstly, MV and/or OPV only prevent measles and/or polio infections. Secondly, once measles and/or polio infections are eradicated MV and/or OPV campaigns can be phased out, saving resources and without any negative implications for child health and survival; phasing out the smallpox vaccine seems to have had an impact on survival in both high [51] and low-income countries [52, 53]. Furthermore, demonstrated beneficial NSEs in RECAMP-OPV will highlight the need to identify alternative ways to keep stimulating the immune system after the discontinuation of OPV in routine vaccination program services.

## Supplementary information

**Additional file 1.** Analysis plan_RECAMP-MV

**Additional file 2.** Analysis plan_RECAMP-OPV

## Data Availability

Upon request to the corresponding authors (only collaboratively).

## Abbreviations

AEFI: Adverse events following immunization
BCG: Bacille-calmette-guerin
BHP: Bandim Health Project
HDSS: Health and demographic surveillance system
MV: Measles vaccination
NSEs: Non-specific effects
OPV: Oral polio vaccination
PYRS: Person-years at risk
RECAMP-MV: Real-life Effect of a CAMPaign with a Measles Vaccine
RECAMP-OPV: Real-life Effect of a CAMPaign with an Oral Polio Vaccine
SAGE: Strategic Advisory Group of Experts on Immunizations
WHO: World Health Organization
