# Supplementary material for: Research protocol of two concurrent cluster-randomized trials: Real-life Effect of a CAMPaign with Measles Vaccination (RECAMP-MV) and Real-life Effect of a CAMPaign with Oral Polio Vaccination (RECAMP-OPV) on mortality and morbidity among children in rural Guinea-Bissau
Source: BMC Public Health. 2019 Nov 11;19:1506. doi: 10.1186/s12889-019-7813-y (PMC6849174; doi:10.1186/s12889-019-7813-y)
Supplement: Supplementary file 1 — Additional file 1. Analysis plan_RECAMP-MV [file 12889_2019_7813_MOESM1_ESM.docx]

# The effect of a Measles Vaccination Campaign on morbidity and mortality among children aged 9-59 months in Rural Guinea-Bissau – a Cluster Randomized Controlled Trial

INDEX

[1. ANALYSES OF BASELINE COMPARABILITY 2](#_Toc485823877)

[**Table S1: Summary of background factors by MVC-group and control group** 2](#_Toc485823878)

[2. PRIMARY ANALYSIS OF PRIMARY OUTCOME 3](#_Toc485823879)

[**Table S2: Primary analysis of primary outcome** 3](#_Toc485823880)

[3. PRIMARY ANALYSES OF SECONDARY OUTCOMES 4](#_Toc485823881)

[**Table S3: Primary analysis of secondary outcome: non-accidental repeated morbidity** 4](#_Toc485823882)

[**Table S4: Primary analysis of secondary outcome: non-accidental mortality** 4](#_Toc485823883)

[4. EFFECT MODIFIER ANALYSES OF THE PRIMARY OUTCOME](#_Toc485823884)

**[Table S5: Effect modifier analysis of primary outcome](#_Toc485823884)**[…………………………………………5](#_Toc485823884)

[5. SECONDARY ANALYSES OF SECONDARY OUTCOMES](#_Toc485823885)

**[Table S6: Secondary analyses of secondary outcome: cause specific primary outcome](#_Toc485823885)** [7](#_Toc485823885)

[**Table S7: Secondary analysis of secondary outcome: first non-accidental outpatient visit** 7](#_Toc485823886)

[**Table S8: Secondary analysis of secondary outcome: first non-accidental illness** 7](#_Toc485823887)

[6. SENSITIVITY ANALYSES OF PRIMARY OUTCOME](#_Toc485823888)

**[Table S9: Sensitivity analysis of primary outcome censoring for measles cases](#_Toc485823888)** [8](#_Toc485823888)

[**Table S10: Sensitivity analysis of primary outcome with intention-to-treat analysis** 8](#_Toc485823889)

# 1. ANALYSES OF BASELINE COMPARABILITY

We will describe baseline characteristics for the MVC-group and the control group. Categorical variables will be presented as frequencies and proportions and continuous variables will be presented as either medians with interquartile ranges or means with standard deviations.

## **Table S1: Summary of background factors by MVC-group and control group**

| - Region - Vaccination coverage - Age - Prior MV status - Sex - Participation in other health interventions prior to enrolment - Season - Weight - Temperature - Mid-upper-arm circumference - Type of acute illness on the day of enrolment - Medicine intake on the day of enrolment - Medicine provided by enrolling nurse - Vaccination card verified - Socioeconomic factors (maternal education and housing conditions) |
| --- |

#

# 2. PRIMARY ANALYSIS OF PRIMARY OUTCOME

## **Table S2: Primary analysis of primary outcome**

| **Type** | per-protocol analysis |
| --- | --- |
| **Population** | children receiving intervention or control assignment as randomly allocated |
| **Censoring** | - death due to accident - migration - trial end |
| **Time scale** | age |
| **Failure** | non-accidental morbidity (first non-fatal hospitalization with overnight stay) OR  non-accidental mortality (death) |
| **Stata code** | *Analysis* stset outdate, f(combined_outcome=1) origin(date_of_birth) enter(date_of_enrolment) /// exit(censoring_date)  stcox group, strata(region vaccination_coverage sex) vce(cl cluster)  (If we in general identify evidence for non-proportionality, we will still report the marginal hazard ratios but supplement this measure by hazard ratios for 2-3 properly selected categorical time-periods identified based on the aforementioned proportionality investigations).  *Check of proportional hazards assumption* estat phtest, detail  stphplot, strata(group) adj(region vaccination_coverage sex)  stcox group, strata(region vaccination_coverage sex) vce(cl cluster) tvc(group) texp(_t)^[[1]](#footnote-1)^ |

#

# **3. PRIMARY ANALYSES OF SECONDARY OUTCOMES**

## **Table S3: Primary analysis of secondary outcome: non-accidental repeated morbidity**

| **Type** | per-protocol analysis |
| --- | --- |
| **Population** | children receiving intervention or control assignment as randomly allocated |
| **Censoring** | - death due to accident - migration - trial end |
| **Time scale** | age |
| **Failure** | non-accidental repeated morbidity (at least one non-fatal hospitalization with overnight stay) |
| **Stata code** | *Analysis* outdate1=date_of_observation_period_end, indate1=date_of_observation_period_beginning  stset outdate1, f(morbidity=1) origin(date_of_birth) enter(date_of_enrolment) /// time0(indate1) exit(censoring_date) id(child_identification_number)  stcox group, strata(region vaccination_coverage sex) vce(cl cluster)  *Check of proportional hazards assumption*  estat phtest, detail  stphplot, strata(group) adj(region vaccination_coverage sex)  stcox group, strata(region vaccination_coverage sex) vce(cl cluster) tvc(group) texp(_t) |

## **Table S4: Primary analysis of secondary outcome: non-accidental mortality**

| **Type** | per-protocol analysis |
| --- | --- |
| **Population** | children receiving intervention or control assignment as randomly allocated |
| **Censoring** | - death due to accident - migration - trial end |
| **Time scale** | Age |
| **Failure** | non-accidental mortality (death) |
| **Stata code** | *Analysis* outdate1=date_of_observation_period_end, indate1=date__of_observation_period_beginning  stset outdate1, f(mortality=1) origin(date_of_birth) enter(date_of_enrolment) /// time0(indate1) exit(censoring_date) id(child_identification_number)  stcox group, strata(region vaccination coverage sex) vce(cl cluster)  *Check of proportional hazards assumption*  estat phtest, detail  stphplot, strata(group) adj(region vaccination_coverage sex)  stcox group, strata(region vaccination_coverage sex) vce(cl cluster) tvc(group) texp(_t) |

4. EFFECT MODIFIER ANALYSES OF THE PRIMARY OUTCOME
Table S5: Effect modifier analysis of primary outcome

| **Type** | per-protocol analysis |
| --- | --- |
| **Population** | children receiving intervention or control assignment as randomly allocated |
| **Censoring** | - death due to accident - migration - trial end |
| **Time scale** | age |
| **Failure** | non-accidental morbidity (first non-fatal hospitalization with overnight stay) OR  non-accidental mortality (death) |
| **Description** | We construct a new four-level variable (group EfM) based on the four possible combinations of group and the effect modifier. |
| **Effect modifier (EfM) in three separate models** | Prior MV status: yes or no to MV before enrolment Sex: male or female Season: rainy season (June-November) or dry season (December-May)   *Individual records will remain as in the primary analysis, and the model allow for interaction with the potential effect modifier.* |
| **Stata code** | *Analysis* Egen group_EfM=group(group EfM)  stset outdate, f(combined_outcome=1) origin(date_of_birth) /// enter(date_of_enrolment) exit (censoring_date)  stcox group#EfM EfM, strata(region vaccination_coverage sex) vce(cl cluster) contrast group#EfM   *Check of proportional hazards assumption* estat phtest, detail  stphplot, strata(group EfM) adj(region vaccination_coverage, sex)  stcox group#EfM EfM, strata(region vaccination_coverage sex) vce(cl cluster) /// tvc(group#EfM EfM) texp(_t) |
| **Effect modifier Vitamin A supplement  (for other campaigns implemented during enrolment e.g. meningitis A, inactivated polio vaccine, the same analysis approach will be applied)** | *Vitamin A supplement: to be analysed as a time varying exposure*  *Analysis* Egen group_vitAsup=group(group vitAsup)  stset outdate, f(combined_outcome=1) origin(date_of_birth) /// enter(date_of_enrolment) exit (censoring_date) id(child_identification_number)  stsplit vitAsup, at(0) after(first_vitAsup)  stcox group#vitAsup vitAsup, strata(region vaccination_coverage sex) ///  vce(cl cluster) contrast group#vitAsup^[[2]](#footnote-2)^  *Check of proportional hazards assumption* estat phtest, detail  stphplot, strata(group vitAsup) adj(region vaccination_coverage, sex) stcox group#vitAsup vitAsup, strata(region vaccination_coverage sex) /// vce(cl cluster) tvc(group#vitAsup vitAsup) texp(_t) |

5. SECONDARY ANALYSES OF SECONDARY OUTCOMES
Table S6: Secondary analyses of secondary outcome: cause specific primary outcome

| **Type** | per-protocol analysis |
| --- | --- |
| **Population** | children receiving intervention or control assignment as randomly allocated |
| **Censoring** | - death due to accident - migration - trial end |
| **Time scale** | age |
| **Failure** | non-accidental morbidity (first non-fatal hospitalization with overnight stay) OR  non-accidental mortality (death) due to: malaria, diarrhea, respiratory infection |
| **Stata code** | *Analysis* stset outdate, f(combined_outcome=1&cause==X) origin(date_of_birth) /// enter(date_of_enrolment) exit(censoring_date)  stcox group, strata(region vaccination_coverage sex) vce(cl cluster)  *Check of proportional hazards assumption* estat phtest, detail  stphplot, strata(group) adj(region vaccination_coverage sex)  stcox group, strata(region vaccination_coverage sex) vce(cl cluster) tvc(group) texp(_t) |

## **Table S7: Secondary analysis of secondary outcome: first non-accidental outpatient visit**

| **Type** | per-protocol analysis |
| --- | --- |
| **Population** | children receiving intervention or control assignment as randomly allocated but 1-2 months after assignment visited to inquire about short-term morbidity in the time that as elapsed since assignment |
| **Count** | proportion of non-accidental outpatient visits in a sub-group |
| **Stata code** | binreg outpatient_visit group b1.reg. b0.vaccination_coverage b1.sex, rr vce(cl cluster) |

## **Table S8: Secondary analysis of secondary outcome: first non-accidental illness**

| **Type** | per-protocol analysis |
| --- | --- |
| **Population** | children receiving intervention or control assignment as randomly allocated but 1-2 months after assignment visited to inquire about short-term morbidity in the time that as elapsed since assignment |
| **Count** | proportion of non-accidental illness in a sub-group |
| **Stata code** | binreg illness group b1.reg. b0.vaccination_coverage b1.sex, rr vce(cl cluster) |

6. SENSITIVITY ANALYSES OF PRIMARY OUTCOME
Table S9: Sensitivity analysis of primary outcome censoring for measles cases

| **Type** | per-protocol analysis |
| --- | --- |
| **Population** | children receiving intervention or control assignment as randomly allocated |
| **Censoring** | - death due to measles infection - death due to accident - migration - trial end |
| **Time scale** | age |
| **Failure** | non-accidental morbidity (first non-fatal hospitalization with overnight stay) OR  non-accidental mortality (death) |
| **Stata code** | *Analysis* stset outdate, f(combined_outcome=1) origin(date_of_birth) enter(date_of_enrolment) /// exit(censoring_date) stcox group, strata(region vaccination_coverage sex) vce(cl cluster)  *Check of proportional hazards assumption* estat phtest, detail  stphplot, strata(group) adj(region vaccination_coverage sex)  stcox group, strata(region vaccination_coverage sex) vce(cl cluster) tvc(group) texp(_t) |

## **Table S10: Sensitivity analysis of primary outcome with intention-to-treat analysis**

| **Type** | classic intention-to-treat analysis (ITT-C) |
| --- | --- |
| **ITT-C population** | including all children present in the village from the day they were first potentially eligible to enter the trial but did not because they e.g. did not receive the assigned treatment, were excluded due to illness, had no guardian present, had a guardian who refused participation |
| **Type** | extended intention-to-treat analysis (ITT-E) |
| **ITT-E population** | including all children living in the village from the day they were first potentially eligible to enter the trial if present and healthy as the intervention may also affect the health of other children in the community by reducing exposure to severe infections (date of entry in analysis=date_of_enrolment_X) |
| **Censoring** | - death due to accident - migration - trial end |
| **Time scale** | age |
| **Failure** | non-accidental morbidity (first non-fatal hospitalization with overnight stay) OR  non-accidental mortality (death) |
| **Stata code** | *Analysis* stset outdate, f(combined_outcome=1) origin(date_of_birth) enter(date_of_enrolment_X) /// exit(censoring_date)  stcox group, strata(region vaccination_coverage sex) vce(cl cluster)  *Check of proportional hazards assumption* estat phtest, detail  stphplot, strata(group) adj(region vaccination_coverage sex)  stcox group, strata(region vaccination_coverage sex) vce(cl cluster) tvc(group) texp(_t) |

1. In supplementary investigations, it can in general be considered to replace the term texp(_t) with texp(_t>s) for specific values of s if relevant after assessing the log-log survival curves. [↑](#footnote-ref-1)
2. VAS campaigns are conducted approximately every 6 months and target all children >6 months old. As children receiving VAS after enrolment will be older than children who have not yet received VAS, disentangling differential MV effects by age, time since intervention and pre/post VAS is problematic. In an attempt to investigate if it is a time since enrolment-/age-differential effect, rather than a differential effect of campaign MV by VAS, we will furthermore explore the effect in models allowing for 3-way interactions between: MVC-group, pre- vs post-VAS campaign and first 3 months after enrolment vs subsequent. To do so, observation time for each child will be split into 2 further time-bands: the first 3 months after enrolment and subsequent months. We will then test if the effect of MVC-group varies in the resulting 4 groups: A) No VAS, <3 months after enrolment; B) No VAS, >3months after enrolment, C) VAS, <3 months after enrolment; D) VAS, >3months after enrolment. [↑](#footnote-ref-2)
