# Supplementary material for: Research protocol of two concurrent cluster-randomized trials: Real-life Effect of a CAMPaign with Measles Vaccination (RECAMP-MV) and Real-life Effect of a CAMPaign with Oral Polio Vaccination (RECAMP-OPV) on mortality and morbidity among children in rural Guinea-Bissau
Source: BMC Public Health. 2019 Nov 11;19:1506. doi: 10.1186/s12889-019-7813-y (PMC6849174; doi:10.1186/s12889-019-7813-y)
Supplement: Supplementary file 2 — Additional file 2. Analysis plan_RECAMP-OPV [file 12889_2019_7813_MOESM2_ESM.docx]

Analysis plan – 22-07-2017

**RECAMP-OPV: A Cluster Randomised Controlled Trial of the effect of Oral Polio Vaccine campaigns on child morbidity and mortality.**

Contents

[1 ANALYSES OF BASELINE COMPARABILITY 3](#_Toc488511674)

[1.1 Participant flow from eligibility to follow-up 3](#_Toc488511675)

[2 PRIMARY ANALYSIS OF PRIMARY OUTCOME 3](#_Toc488511676)

[3. PRIMARY ANALYSES OF SECONDARY OUTCOMES 4](#_Toc488511677)

[We have defined the primary outcome as a composite outcome to have sufficient power to obtain an answer before the global withdrawal of OPV. However, we will also investigate the effect of OPV on either of the components. 4](#_Toc488511678)

[3.1 All-cause mortality 4](#_Toc488511679)

[3.2 All cause morbidity 5](#_Toc488511680)

[4 SECONDARY ANALYSES OF PRIMARY OUTCOME 6](#_Toc488511681)

[4. 1 Secondary analyses of primary outcome – Effect modifiers 6](#_Toc488511682)

[4.2 Secondary analyses of primary outcome – Intention-to-treat analyses 8](#_Toc488511683)

[5 SECONDARY ANALYSES OF SECONDARY OUTCOMES 8](#_Toc488511684)

[5.1 Cause specific severe morbidity/death based on a structured interview 8](#_Toc488511685)

[5.2 Short term morbidity and growth 9](#_Toc488511686)

[6 SENSITIVITY ANALYSES 9](#_Toc488511687)

[7 REFERENCES 10](#_Toc488511688)

# 1 ANALYSES OF BASELINE COMPARABILITY

## 1.1 Participant flow from eligibility to follow-up

Children aged 0-8 months living with registered families in the Bandim Health Project’s Rural Health and Demographic Surveillance System will be eligible to enter this trial. On the enrolment day, the nurse will exclude children from entering the trial if the child fulfills one of the following exclusion criteria:

- the child has a temperature >39°C or is overtly ill
- the child has a mid-upper-arm-circumference <110 mm and age >6 months
- the child has experienced an allergic reaction after a prior vaccination
- the child is aged <2 months and enrolled in a randomised trial of BCG and OPV given at a home visit shortly after birth

**Table S1: Summary of background factors by intervention and control group**

| - Sex - Age - Region - Weight - Temperature - Mid-upper-arm circumference - Symptoms on the day of enrolment - Medicine intake on the day of enrolment - Provided with medicine by enrolling nurse - Participation in other health interventions prior to enrolment - Vaccination status - Breastfeeding status - Socioeconomic factors (maternal education and housing conditions) |
| --- |

# 2 **PRIMARY ANALYSIS OF PRIMARY OUTCOME**

The primary outcome is a composite outcome “severe morbidity” defined as morbidity causing hospital admission or death. Since it can be difficult to distinguish between admissions and outpatient visits through interviews, we have defined admission as an overnight stay in a health facility. Hospital admissions due to accidents do not count as events, but the follow-up time is (interval) censored while the child is admitted. Children who die due to accidents are censored at date of death.

Outcomes will be analysed in regression models based on the individual level data, which is most efficient when the cluster-size varies. For the primary outcome, we will use Cox proportional hazards models, stratified by region, pre-trial vaccination coverage and sex with age as the underlying timescale, cluster-robust standard error to account for intra-cluster correlation. All analyses will be based on 5% significance level and 95% confidence interval.

The per-protocol analysis is the primary analysis. In the per-protocol analysis, children will enter the analysis on the date of enrolment and be followed for up to 12 months to an event (death or first hospital admission) until the next general vaccination campaign for which the child is eligible, migration or end of study whichever comes first. Deaths/hospital admissions due to accidents will be censored.

**Table S2. Primary analysis of primary outcome**

| Type | Per-protocol analysis |
| --- | --- |
| Population | Randomised children receiving assigned treatment |
| Censoring | - Eligibility for vaccination campaign - 12 months of follow-up - Death due to accident - Migration - Trial end |
| Time scale | Age |
| Failure | First hospital admission with overnight stay not due to accident or death due to any non-accident cause |
| Stata code | Analysis:  stset outdate, f(combinedoutcome=1) origin(datebirth) enter(dateenrol) exit(dateenrol+365.25) stcox group, strata(reg vaccov sex) vce(cl cluster)  Check of proportional hazards assumption^[[1]](#footnote-1)^:  estat phtest, detail  stphplot , strata(group) adj((reg vaccov sex)  stcox group, strata((reg vaccov sex) vce(cl cluster) tvc(group) texp(_t)^[[2]](#footnote-2)^ |

# **3. PRIMARY ANALYSES OF SECONDARY OUTCOMES**

We have defined the primary outcome as a composite outcome to have sufficient power to obtain an answer before the global withdrawal of OPV. However, we will also investigate the effect of OPV on either of the components.

## 3.1 All-cause mortality

**Table S3: All-cause mortality**

| Type | Per-protocol analysis |
| --- | --- |
| Population | Randomised children receiving assigned treatment |
| Censoring | - Eligibility for vaccination campaign - 12 months of follow-up - Death due to accident - Migration - Trial end |
| Time scale | Age |
| Failure | Death due to any non-accident cause |
| Stata code | Analysis:  stset outdate, f(death=1) origin(datebirth) enter(dateenrol) exit(dateenrol+365.25) stcox group, strata(reg vaccov sex) vce(cl cluster)  Check of proportional hazards assumption:  estat phtest, detail  stphplot , strata(group) adj((reg vaccov sex)  stcox group, strata((reg vaccov sex) vce(cl cluster) tvc(group) texp(_t) |

## 3.2 All cause morbidity

**Table S4: All-cause admissions**

| Type | Per-protocol analysis |
| --- | --- |
| Population | Randomised children receiving assigned treatment |
| Censoring | - Eligibility for vaccination campaign - 12 months of follow-up - Death - Migration - Trial end |
| Time scale | Age |
| Failure | Any hospital admission visit with overnight stay not due to accident |
| Stata code | Outdate1=date of end of observation period, indate=date of beginning of observation period  Analysis:  stset outdate1, f(admitted=1) origin(datebirth) enter(dateenrol) time0(indate) exit(dateenrol+365.25) id(regidc)  stcox group, strata(reg vaccov sex) vce(cl cluster)  Check of proportional hazards assumption:  estat phtest, detail  stphplot , strata(group) adj((reg vaccov sex)  stcox group, strata((reg vaccov sex) vce(cl cluster) tvc(group) texp(_t) |

# **4 SECONDARY ANALYSES OF PRIMARY OUTCOME**

The secondary analyses of the primary outcome aim to assess if the effect of the OPV campaign varies with potential effect modifiers identified as important in prior studies. Furthermore, the effect is assessed in intention-to-treat analyses.

4. 1 Secondary analyses of primary outcome – Effect modifiers

In per-protocol analyses of the primary outcome, we will assess the effect of the following potential effect modifiers allowing the effect of the intervention to vary with:

- Sex: Previous studies have demonstrated that the effect of OPV is stronger in boys than girls[^1-3^](#_ENREF_1).
- One vs two doses. Observational studies indicate that additional doses of OPV offer additional benefits[^2^](#_ENREF_2). We will investigate if the effect is the same in children seen at two visits one month apart (both in intervention and control villages) and among children who are only seen at the enrolment post once.
- Age at OPV-0: A prior study has indicated that the effect of subsequent vaccines may vary with the age at which the gut was primed[^4^](#_ENREF_4). OPV-0 should be provided with BCG at birth, but is often given later. We will investigate if the effect varies by age of reception of OPV-0 within the first 2 weeks of life vs. after.
- Season: effect in children enrolled in the dry season (December-May) versus children enrolled in the rainy season (June-November)
- Prior OPV campaign: If OPV campaigns take place during the study, we will assess whether the effect is similar in children having been exposed/not exposed to prior OPV campaigns.
- Vitamin A campaigns: Splitting the follow-up time at first vitamin A campaign for which the child is eligible

Except for vitamin A campaigns (to be analysed as a time varying exposure), the individual records will remain as in the primary analysis, and the model allow for interaction with the potential effect modifier.

**Table S5: Effect modifiers**

| Type | Per-protocol analysis |
| --- | --- |
| Population | Randomised children receiving assigned treatment |
| Censoring | - Eligibility for vaccination campaign - 12 months of follow-up - Death due to accident - Migration - Trial end |
| Time scale | Age |
| Failure | First hospital admission with overnight stay not due to accident or death due to any non-accident cause |
| **Effect modifiers (EfM) in 5 separate models)** | Sex: (M/F)  1 or 2 doses: Children seen at two subsequent village one month apart (yes/no)  Age at OPV-0: <15 days; >=15 days or not yet received  Season: Rainy (June-November) or Dry (December-May)  Prior OPV: Eligibility to OPV campaign |
| Stata code | Analysis:  stset outdate, f(combinedoutcome=1) origin(datebirth) enter(dateenrol) exit(censoring) stcox group#EfM EfM, strata(reg vaccov sex) vce(cl cluster)  contrast group#EfM  Check of proportional hazards assumption:  egen group_EfM=group(group EfM)  estat phtest, detail  stphplot , strata(group_EfM) adj((reg vaccov sex)  stcox group#EfM EfM, strata((reg vaccov sex) vce(cl cluster) tvc(group#EfM EfM) texp(_t) |
| **VAS as effect modifier** | Vitamin A supplementation (VAS): VAS campaigns are conducted approximately every 6 months and target all children >6 months old.^[[3]](#footnote-3)^ |
| Stata code | Analysis:  stset outdate, f(combinedoutcome=1) origin(datebirth) enter(dateenrol) exit(dateenrol+365.25) id(regidc)  stsplit VAS, at(0) after(firstVAScampaign)  stcox group#VAS VAS, strata(reg vaccov sex) vce(cl cluster)  contrast group#VAS  Check of proportional hazards assumption:  estat phtest, detail  egen group_VAS=group(group VAS)  stphplot, strata(group_VAS) adj((reg vaccov sex)  stcox group#VAS VAS, strata((reg vaccov sex) vce(cl cluster) tvc(group#VAS VAS) texp(_t) |

## 4.2 Secondary analyses of primary outcome – Intention-to-treat analyses

We will report two types of intention-to-treat analyses:

- Classic ITT analysis: all children who could have entered the trial if heathy and accepted to participate.
- Extended ITT analysis: all children in intervention and control villages from the day they could have been enrolled if home and healthy, as the intervention may also affect the health of other children in the community by reducing exposure to severe infections.

**Table S6: Intention-to-treat analyses**

| Type | Intention-to-treat analysis (ITT) |
| --- | --- |
| Population  (ITT-1) | Classic ITT analysis: Enrolled children + children aged 0-8 months, registered in the HDSS and present at date of enrolment but not enrolled due to not fulfilling health criteria or nor consent to participate. Date of entry: DatenrolX=date when first present at a village visit to enroll children. |
| Population  (ITT-2) | Extended ITT analysis: All children aged 0-8 months, registered in the HDSS and could potentially have entered the study. Date of entry: DatenrolX=date when first attempted visited (alive and registered in village) at a village visit to enroll children. |
| Censoring | - Eligibility for vaccination campaign - 12 months of follow-up - Death due to accident - Migration - Trial end |
| Time scale | Age |
| Failure | First hospital admission with overnight stay not due to accident or death due to any non-accident cause |
| Stata code | Analysis:  stset outdate, f(combinedoutcome=1) origin(datebirth) enter(dateenrolX) exit(dateenrol+365.25) stcox group, strata(reg vaccov sex) vce(cl cluster)  Check of proportional hazards assumption:  estat phtest, detail  stphplot , strata(group) adj((reg vaccov sex)  stcox group, strata((reg vaccov sex) vce(cl cluster) tvc(group) texp(_t) |

# 5 SECONDARY ANALYSES OF SECONDARY OUTCOMES

## 5.1 Cause specific severe morbidity/death based on a structured interview

**Table S7: Cause-specific severe morbidity**

| Type | Per-protocol analysis |
| --- | --- |
| Population | Randomised children receiving assigned treatment |
| Censoring | - Eligibility for vaccination campaign - 12 months of follow-up - Death due to accident - Migration - Trial end |
| Time scale | Age |
| Failure | Health system visit with overnight stay or death due to: Malaria, Respiratory infection, Diarrhea |
| Stata code | stset outdate, f(combinedoutcome==1&cause==X) origin(datebirth) enter(dateenrol) exit(censoring) stcox group, strata(reg vaccov sex) vce(cl cluster)  Check of proportional hazards assumption:  estat phtest, detail  stphplot , strata(group) adj((reg vaccov sex)  stcox group, strata((reg vaccov sex) vce(cl cluster) tvc(group) texp(_t) |

## 5.2 Short term morbidity and growth

For children visited one month after the enrolment visits, the mother/guardian is interviewed on illness and consultations in the period since enrolment. The child is then sent to the vaccination post for health exam and weighing (both trial arms) and second dose of OPV (campaign arm).

**Table S8: Short term morbidity and growth**

| Type | Per-protocol analysis |
| --- | --- |
| Population | Randomised children receiving assigned treatment at enrolment visit |
| Outcomes | Proportion reporting illness in the month after enrolment  Proportion having sought consultations in the month after enrolment  Weight-for-age at one month follow-up adjusted for baseline weigh-for-age  Mid-upper-arm circumference at one month follow-up adjusted for baseline mid-upper-arm circumference |

# 6 SENSITIVITY ANALYSES

In sensitivity analyses, we will assess whether the conclusions are altered by the following censoring criteria:

- Censoring follow-up at 9 months of age when children become eligible for measles vaccine.
- Censoring at other general health intervention campaigns (vitamin A supplementation, intermittent malaria treatment, bednets etc).
- Censoring at registration of subsequent routine vaccines.
- Censoring at scheduled ages for subsequent vaccines.
- To assess whether the effect is changing over time, we will split follow-up time 6 months after enrolment and assess the effect in the two time bands.
- To assess whether the effect varies over the year, the follow-up time will be divided into dry (December-May) and rainy (June-November) season.

# 7 REFERENCES

1. Lund N, Andersen A, Hansen AS, et al. The Effect of Oral Polio Vaccine at Birth on Infant Mortality: A Randomized Trial. *Clinical infectious diseases : an official publication of the Infectious Diseases Society of America* 2015; **61**(10): 1504-11.

2. Andersen A, Fisker AB, Rodrigues A, et al. National immunization campaigns with oral polio vaccine (OPV) reduce the general all-cause mortality rate: An analysis of the effect of campaign-OPV on child mortality within seven randomised trials (submitted). 2017.

3. Andersen A, Bjerregaard-Andersen M, Rodrigues A, Umbasse P, Fisker AB. Sex-differential effects of diphtheria-tetanus-pertussis vaccine for the outcome of paediatric admissions? A hospital based observational study from Guinea-Bissau (under review). 2017.

4. Aaby P, Andersen A, Martins CL, et al. Does oral polio vaccine have non-specific effects on all-cause mortality? Natural experiments within a randomised controlled trial of early measles vaccine. *BMJ open* 2016; **6**(12): e013335.

1. If we identify evidence for non-proportionality, we will still report the marginal hazard ratios, but supplement this estimate by hazard ratios for 2-3 properly selected categorical time-periods identified based on the aforementioned proportionality investigations [↑](#footnote-ref-1)
2. It there is indication of non-proportionality based on assessment of the log-log curves, we will test for non-proportionality by replacing the term texp(_t) with texp(_t>s) for specific values of s. [↑](#footnote-ref-2)
3. As children receiving VAS after enrolment will be older than children who have not yet received VAS, disentangling differential MV effects by age, time since intervention and pre/post VAS is problematic. In an attempt to investigate if it is a time since enrolment-/age-differential effect, rather than a differential effect of campaign MV by VAS, we will furthermore explore the effect in models allowing for 3-way interactions between: MVC-group, pre- vs post-VAS campaign and first 3 months after enrolment vs subsequent. To do so, observation time for each child will be split into 2 further time-bands: the first 3 months after enrolment and subsequent months. We will then test if the effect of MVC-group varies in the resulting 4 groups: A) No VAS, <3 months after enrolment; B) No VAS, >3months after enrolment, C) VAS, <3 months after enrolment; D) VAS, >3months after enrolment. [↑](#footnote-ref-3)
